# Pilot randomized controlled trial of a complex intervention for diabetes self-management supported by volunteers, technology, and interprofessional primary health care teams

**DOI:** 10.1186/s40814-019-0504-8

**Published:** 2019-10-27

**Authors:** Gina Agarwal, Jessica Gaber, Julie Richardson, Dee Mangin, Jenny Ploeg, Ruta Valaitis, Graham J. Reid, Larkin Lamarche, Fiona Parascandalo, Dena Javadi, Daria O’Reilly, Lisa Dolovich

**Affiliations:** 1 Department of Family Medicine, McMaster University, David Braley Health Sciences Centre, 5th Floor, 1280 Main Street West, Hamilton, Ontario L8S 4K1 Canada; 2 School of Rehabilitation Science, McMaster University, 1280 Main Street West, Hamilton, Ontario L8S 4K1 Canada; 3 Department of Health, Aging and Society, Faculty of Health Sciences, McMaster University, 1280 Main Street West, Hamilton, Ontario L8S 4K1 Canada; 4 School of Nursing, McMaster University, 1280 Main Street West, Hamilton, Ontario L8S 4K1 Canada; 5 Departments of Psychology, Family Medicine, & Paediatrics, The University of Western Ontario, Westminster Hall, Room 319E, London, Ontario N6A 3K7 Canada; 6 Department of Health Research Methods, Evidence, and Impact, McMaster University, CRL, 2nd Floor, 1280 Main Street West, Hamilton, Ontario L8S 4K1 Canada

**Keywords:** Primary care, Diabetes, Chronic conditions, Self-management, eHealth, Feasibility trial, Health care volunteers, Hypertension

## Abstract

**Background:**

Most health care for people with diabetes occurs in family practice, yet balancing the time and resources to help these patients can be difficult. An intervention empowering patients, leveraging community resources, and assisting self-management could benefit patients and providers. Thus, the feasibility and potential for effectiveness of “Health Teams Advancing Patient Experience, Strengthening Quality through Health Connectors for Diabetes Management” (Health TAPESTRY-HC-DM) as an approach supporting diabetes self-management was explored to inform development of a future large-scale trial.

**Methods:**

Four-month pilot randomized controlled trial (RCT), sequential explanatory qualitative component. Participants—patients of an interprofessional primary care team—were over age 18 years, diagnosed with diabetes and hypertension, and had Internet access and one of the following: uncontrolled HbA1c, recent diabetes diagnosis, end-stage/secondary organ damage, or provider referral. The Health TAPESTRY-HC-DM intervention focused on patient health goals/needs, integrating community volunteers, eHealth technologies, interprofessional primary care teams, and system navigation. Pilot outcomes included process measures (recruitment, retention, program participation), perceived program feasibility, benefits and areas for improvement, and risks or safety issues. The primary trial outcome was self-efficacy for managing diabetes. There were a number of secondary trial outcomes.

**Results:**

Of 425 eligible patients invited, 50 signed consent (11.8%) and 35 completed the program (15 intervention, 20 control). Volunteers (*n* = 20) met 28 clients in 234 client encounters (home visits, phone calls, electronic messages); 27 reports were sent to the interprofessional team. At 4 months, controlling for baseline, most outcomes were better in the intervention compared to control group; physical activity notably better. The most common goal domains set were physical activity, diet/nutrition, and social connection. Clients felt the biggest impact was motivation toward goal achievement. They struggled with some of the technologies. Several participants perceived that the program was not a good fit, mostly those that felt they were already well-managing their diabetes.

**Conclusions:**

Health TAPESTRY-HC-DM was feasible; a large-scale randomized controlled trial seems possible. However, further attention needs to be paid to improving recruitment and retention. The intervention was well received, though was a better fit for some participants than others.

**Trial registration:**

ClinicalTrials.gov, NCT02715791. Registered 22 March 2016—retrospectively registered.

## Background

Diabetes is a chronic condition, with serious complications if not managed appropriately [1, 2]. The development and improvement of diabetes are associated with lifestyle, so patient behavior and empowerment are important in management. Patients who are more engaged in self-management of their diabetes have better short- and long-term outcomes [3]. Diabetes self-management support is an ongoing process which can include behavioral, educational, and psychosocial methods to facilitate the knowledge, skills, ability, and methods a person with diabetes can use to manage their condition and sustain healthy behaviors [4, 5]. In several systematic reviews and meta-analyses, diabetes self-management programs have led to positive outcomes including improvements in glycemic control, HbA1c, self-efficacy, diabetes knowledge, and self-management behaviors [3, 6–8].

The majority of care for people with diabetes occurs in primary care. In Ontario, Canada, approximately three quarters of people with diabetes see only a family physician for care [9, 10]. However, collaborative care, including interprofessional primary care teams supported by diabetes specialists, is recognized as the standard of optimal care for diabetes management [11]. Such teams are well positioned to empower, engage, and motivate patients with their diabetes self-management. Person-focused care is another core feature of primary care. It involves patients and primary care providers working as partners in health care and shared decision making [12]. However, providers face challenges in balancing the time, financial resources, and support required to care for their patients with diabetes and being able to fully partner in healthy lifestyle self-management [10]. Therefore, an intervention that can facilitate self-management by employing technology people can use at home and leveraging community resources could be beneficial for patients, providers, and other caregivers.

In response to these challenges, we developed “Health Teams Advancing Patient Experience, Strengthening Quality through Health Connectors for Diabetes Management” (Health TAPESTRY-HC-DM). The Health TAPESTRY-HC-DM approach centers on patients’ prioritized health goals and needs and aims to build capacity for diabetes and other chronic condition self-management. The Health TAPESTRY-HC-DM intervention adapted the multi-component Health TAPESTRY approach [13–15] to specifically focus on fostering self-management for persons with diabetes and hypertension. Health TAPESTRY-HC-DM supports patients through the integration of several components: (i) trained community volunteer “Health Connectors” who facilitate goal setting and motivation, connect clients to local community programs, and provide resources and tech support; (ii) technology including the use of a web-based data collection and self-management application and a personal health record; (iii) the interprofessional primary health care team; and (iv) engagement with community programs, with system navigation enhanced through all of these components.

Trained volunteers can enhance care delivery as extensions of primary health care teams, in a manner that is structured and low risk to both patients and volunteers. When community members in health-connecting roles like community health workers or peer volunteers are integrated into interventions for diabetes care, research has shown many positive outcomes for the patients such as improved knowledge about diabetes, patient activation and diabetes self-efficacy, glycemic control, health behaviors (including diet, physical activity, and self-monitoring of blood glucose), and quality of life, and decreased diabetes distress, HbA1c, body mass index, and blood pressure [16–20].

Technology is also an accepted tool in health care delivery. Enhancing care through diabetes-specific eHealth innovations can be beneficial. Emerging literature suggests that virtual environments can provide a useful platform for diabetes education and self-management support for people with diabetes [21]. In a 2013 review including 104 studies (62 randomized controlled trials [RCTs], 42 not defined), 74% of studies showed some form of added benefit to diabetes management with technology use [22]; a 2011 review of nine RCTs found that HbA1c decreased in patients who used web-based tools in particular, though they were not effective in reducing fasting plasma glucose [23].

Another aspect that can be integrated into healthy lifestyle self-management is participation in community programs, which offers a multitude of potentially untapped resources. Raising awareness of and access to these community-based health and social service programs have the potential to enhance patient care.

As recommended in clinical guidelines for diabetes care [11], the chronic care model (CCM) was used in developing the Health TAPESTRY intervention [13]. The fundamental assumption of the CCM is that improving care for chronic disease requires connection and collaboration between patient-, provider-, and system-level interventions [24]. The model incorporates six elements which were initially used in developing Health TAPESTRY-HC-DM to identify key components to incorporate into the intervention to ensure success in addressing the burden of multimorbidity beyond solely the patient and health care provider. The Health TAPESTRY-HC-DM intervention worked from a person-focused approach, starting from patients’ needs and experiences within the three “overlapping galaxies” of the CCM: the health care system, the provider organization, and the larger community [25].

The overall aim of this study was to explore the feasibility and signals of effectiveness of the Health TAPESTRY-HC-DM approach in supporting the self-management of chronic conditions, to inform the development of a large-scale future randomized controlled trial. Pilot outcomes included process measures (recruitment, retention, and program participation), perceived program feasibility, benefits and areas for improvement of the program, and any risk or safety issues. The proposed primary trial outcome was self-efficacy for managing diabetes at 4 months in the Health TAPESTRY-HC-DM intervention group compared with the control group; there were several secondary trial outcomes. Feasibility criteria for all proposed trial outcomes were completion rates of outcome measures, estimates of treatment effect, and variance of treatment effect.

## Methods

### Design

We conducted an unblinded pilot randomized controlled trial (RCT) with a sequential explanatory qualitative component. Participants were randomly allocated to the Health TAPESTRY-HC-DM intervention or to a wait list control group. The control group received usual care, followed by the elements of the intervention that were feasible to include after the 4-month intervention period (see the “Control group” section). There were several reasons for conducting this feasibility study, including identifying the feasibility of processes such as recruitment, retention, and participation of patients, volunteers, and health care providers; understanding challenges in survey completion and implementation of the intervention; and estimating the effects and variance of the intervention. Our reporting was guided by the CONSORT checklist for pilot and feasibility trials (see Additional file 1) and the COREQ checklist for reporting qualitative research (see Additional file 2) and registered on ClinicalTrials.gov (NCT02715791).

### Setting

This study was conducted with the McMaster Family Health Team (MFHT) in Hamilton, Ontario, Canada, and involved their patients from Hamilton and surrounding communities. During the time of the study, the MFHT had approximately 32,000 rostered patients, 21 full-time equivalent family physicians, 74 family medicine residents, 18 nurses including 11 full- and part-time nurse practitioners, and 26 allied healthcare professionals at two practice sites. The MFHT is supported by an electronic medical record (EMR).

### Participants

Participants were patients rostered to MFHT who were over the age of 18 years, diagnosed with type 2 diabetes and hypertension, and had access to a computer or smartphone with Internet. They also had to have at least one of the following: recent diagnosis of type 2 diabetes (within the last 6 months); uncontrolled HbA1c (10.0% or higher within the last 6 months or nearest measurement); end-stage or secondary organ damage or complications associated with diabetes (e.g., renal dysfunction, diabetic neuropathy); or referral by a health care provider to the program. We excluded patients who were residents in long-term care, receiving palliative or end-of-life care, participants of previous Health TAPESTRY projects, or having explicitly stated disinterest in research projects to the clinical team.

### Recruitment

The MFHT consented to participate in the study. Individual family physicians had the ability to opt out of the study. We extracted an initial list of participating physicians’ patients with both diabetes and hypertension using the EMR, with chart audit verification; family physicians then reviewed these patient lists for the exclusion criteria. We mailed information and consent packages to patients remaining on the list, and research staff followed up with phone calls. Participants were recruited from January to June 2016, at which point recruitment was ended based on feasibility of study timelines, funding, and staffing.

### Intervention

The intervention period was 4 months. Each client (participant) was set up with a pair of trained community volunteer “Health Connectors.” Health Connectors were recruited by a local community volunteer agency and trained by the program team through a standardized multi-modal training program including in-person and online training and a paper manual, developed based on evidence and expert opinion from program implementation and health care professionals. The volunteer agency set up volunteer pairs with clients and managed the initial home visit scheduling. Health Connectors then conducted regular client encounters through home visits, phone calls, or secure messaging via kindredPHR™ (personal health record). In the first home visit, Health Connectors helped the client to log onto the online Healthy Lifestyle App (described below) and kindredPHR and set goals for healthy lifestyle behavior change. Over the next 4 months (the intervention period), Health Connectors communicated with their clients approximately weekly. During these client encounters, the volunteers worked with their clients as needed by providing motivation for behavior change, access to resources, technology support, and connections to community or clinical programs.

The Healthy Lifestyle App is a web-based tool specially designed for data collection and resource provision. App development was informed by a usability study which found that overall, clients had a positive experience using the app; suggestions for improvement to the layout and content of the app from the usability study were communicated to the design team and implemented prior to the launch of this pilot RCT [26]. The app includes multiple modules with self-assessment surveys and tip sheets (see Table 1). Clients completed the modules at home, with the facilitation of volunteer Health Connectors where desired. The information gathered on the app was organized into client reports which included client goals, key information, survey response summaries, and suggested tip sheets. The reports were securely sent electronically to the client, their volunteers, and their family physician and reviewed during existing weekly interprofessional “huddle” teams at each clinic site which managed intake and care coordination of the reports.

**Table 1:** List of Healthy Lifestyle App modules and tip sheets

Healthy Lifestyle App module	Associated tip sheets based on responses
Diabetes	Blood glucose log
	Community programs for fitness and nutrition
	Complications of diabetes
	Diabetes and eye care
	Diabetes and food care
	Lessening the pain from fingertip testing
	Lows and highs—blood glucose levels
	Managing weight
	Managing your blood glucose
	Nutrition for people with diabetes
	Physical activity for people with diabetes
	Setting reminders for checking your blood glucose levels
	What A1C should I target
Exercise	Community programs for fitness and nutrition
	Flexibility exercises when sitting
	Flexibility exercises when standing
	Muscle strengthening activities
Goal Setting	None
Hypertension	Complications related to hypertension
	DASH diet
	Managing your blood pressure
	Monitoring your blood pressure at home
	Physical activity for people with hypertension
	Why to monitor cholesterol
Medications	Introduction to RxISK.org
	Over-the-counter pain medication tips
	Tips to remember to take medications
Nutrition	DASH diet
	Nutrition for people with diabetes
Personal health record	kindredPHR user manual
Sleep	Good sleep habits—tips for an improved sleep
	Nocturia or frequent urination at night
	Relaxation exercises for falling asleep
	Tips for over-the-counter sleep aids
	When to see your doctor for sleep-related issues

Huddle teams consisted of a range of health professionals including dietitians, occupational therapists, system navigators, pharmacists, registered practical nurses and nurse practitioners, and physiotherapists. They reviewed the reports from their interprofessional lenses and then made appointments or referrals as needed based on patient-identified health goals or needs.

### Randomization and masking

Randomization was conducted through an automated, central (allocation concealed) computerized randomization sequence set up by a statistician on the project who was not involved in data collection and implemented in REDCap [27]. The patient was the unit of randomization. Randomization was stratified by MFHT site and used balanced block allocation.

Participants were aware of their group allocation as the intervention group received the intervention immediately while the control was offered intervention elements after 4 months. Providers knew a patient was receiving the intervention once a Health TAPESTRY-HC-DM report was received, but were not informed of actual group allocation. Research staff were not blinded and had access to files identifying participant study group allocation status, as they had to coordinate with the volunteer agency to ensure participants started the intervention at the appropriate point.

### Control group

The control group received usual care. These patients did not have volunteer visits, did not use the Healthy Lifestyle App, and were not discussed at huddles based on their participation in the program. At the end of the 4-month control period, the control group could opt in to receive some or all of the intervention. All control participants were offered use of kindredPHR and the Healthy Lifestyle App, with any completed reports going to the huddle teams. Control participants who reached their 4-month mark during the contract period of the volunteer coordination agency were also offered connections to volunteer Health Connectors.

### Outcomes and measures

#### Pilot outcomes

Pilot outcomes included process measures (the proportion of family practices that participated, number of patients generated by EMR query, number of patients excluded based on inclusion and exclusion criteria [with reasons for exclusion], number of participants recruited, appropriateness of randomization process, number of participants who withdrew [with reasons for withdrawal], number of participants who completed the intervention, proportion of participants who completed each Healthy Lifestyle App survey, number of volunteers recruited, number of volunteers trained, number and type of client encounters made by volunteers, and number of reports sent to the clinic and seen by the interprofessional huddle team); perceived program feasibility; benefits and areas for improvement of the program; and any risk or safety issues arising from this pilot. The primary data collection method, i.e., the Healthy Lifestyle App, was already pilot tested with results presented in a previous publication [26]. See Table 2 for a complete list of outcomes and data sources.

**Table 2:** Outcomes, timeline, and data sources

Pilot outcomes			
Process measures of recruitment, retention, and program participation		Data source	
Proportion of family practices that participated		Response via paper patient list	
Number of patients generated in EMR query		EMR Query output (Excel document)	
Number of patients excluded based on inclusion and exclusion criteria (with reasons for exclusion)		Research files—data from provider exclusion, chart audit exclusion, or exclusion based on phone conversation with patient	
Number of participants recruited		Research files	
Appropriateness of randomization process		Research files	
Number of participants who withdrew (with reasons for withdrawal)		Research files—data from clients	
Number of participants who completed the intervention		Research files	
Proportion of participants who completed each Healthy Lifestyle App survey		Healthy Lifestyle App	
Questions missed in completed Healthy Lifestyle App surveys		Healthy Lifestyle App	
Number of volunteers recruited		Volunteer agency files	
Number of volunteers trained		Volunteer agency files	
Number of client encounters made by volunteers, and type		Healthy Lifestyle App	
Number of reports sent to the clinic and seen by the interprofessional huddle team		Research tracking based on reports created from Healthy Lifestyle App data and sent to EMR	
Other pilot outcomes		Data source	
Perceived program feasibility		Qualitative interviews	
Risks or safety issues arising from this pilot		Qualitative interviews	
Trial outcome assessment			
Outcome	Outcome measure	Time collected	Data source
Diabetes self-efficacy	Stanford Diabetes Self-Efficacy Scale [28]	T_0_, T_4_	In-person sessions with research staff
	Eight items, ranges from 1 to 10, higher scores indicate better self-efficacy for managing diabetes		
Self-efficacy in managing chronic disease	Stanford self-efficacy for managing chronic disease [29]	T_0_, T_4_	In-person sessions with research staff
	6 items, score ranges from 1 to 10, higher scores indicate better self-efficacy for managing chronic diseases		
Readiness to change	Readiness to change questionnaire (based on a cardiovascular version [30])	T_0_, T_4_	In-person sessions with research staff
	3 items, score ranges from 1 to 5, lower scores indicate higher readiness to change		
Physical activity	Rapid Assessment of Physical Activity (RAPA)—Aerobic Sub-scale [31]	T_0_, T_4_	For T_0_ baseline intervention: Healthy Lifestyle app
	7 items, score ranges from 1 to 7, higher scores indicate more physical activity (< 6 indicates suboptimal activity)		For control group and T_4_: in-person sessions with research staff
HbA1c	EMR Chart Audit	T_−12_, T_0_, T_4,_ T_10_*	EMR
Perceived patient empowerment	Patient Empowerment [32]	T_0_, T_4_	In-person sessions with research staff
	5 items, score ranges from 1 to 4, higher scores represent perceiving more empowerment from the health care team		
Patient-centeredness that participants perceive of their primary care clinic	Patient-centeredness [32]	T_0_, T_4_	In-person sessions with research staff
	6 items, score ranges from 1 to 4, higher scores represent perceiving the health care team as more patient-centered		
Satisfaction with healthcare	Patient Assessment of Chronic Illness Care (PACIC) [33]	T_0_, T_4_	In-person sessions with research staff
	20 items, score ranges from 1 to 10, higher scores represent more positive assessment of care		
Attainment of health goals	Goal attainment scaling	T_4_	In-person sessions with research staff
	Score ranges from − 10 to 110, with higher scores indicating better perception of goal attainment		
Qualitative data			
Measure	Time collected	Data source	
Qualitative patient interviews	T_4_	In-person interview with research staff	

*Timelines were not always possible due to the constraints of using existing EMR chart data, so the closest available readings were included

*EMR* electronic medical record

#### Trial outcome assessment

The proposed primary trial outcome was self-efficacy for managing diabetes at 4 months in the Health TAPESTRY-HC-DM intervention group, compared with the control group. This was measured using the Self-Efficacy for Diabetes scale. This eight-item survey assesses confidence in carrying out various diabetes self-management tasks; items are rated on a 10-point scale, with higher values indicating better self-efficacy for managing diabetes [28]. This survey is validated in this population; internal consistency for the survey for this study was satisfactory (*α* = 0.83).

Proposed secondary outcomes included patient-reported outcome measures (PROMs), patient-reported experience measures (PREMs) [34], and goal attainment scaling (GAS) (see Table 2 for further detail). The PROMs tested were self-efficacy for managing chronic disease [29]; readiness-to-change for three diabetes management-related behaviors (general diabetes management, physical activity, and diet) adapted from a cardiovascular version [30] and based on the Transtheoretical Model of Behaviour Change [35]; self-reported amount and intensity of average weekly physical activity using the Rapid Assessment of Physical Activity [RAPA] [31]; and HbA1c, found through an EMR chart audit. The PREMs tested were patient empowerment, patient centeredness [32], and patient assessment of chronic illness care (PACIC) [33]. Finally, goal attainment scaling was used to gauge clients’ perceived attainment of their top three goals. Domains of goals set were also tracked descriptively, based on the domains created in previous Health TAPESTRY studies [36], with two changes for this population based on a smaller Health TAPESTRY-HC-DM pilot: an additional goal domain of “lose weight” and the medical domain split into “diabetes management” and “medical (other than diabetes management)”.

The feasibility criteria for all proposed trial outcomes were completion rates of outcome measures, missing data, and estimates of treatment effect, and variance of treatment effect.

### Interviews

We collected qualitative data via patient interviews to contribute to the understanding of the feasibility of implementation of a larger trial. Volunteer focus groups were also conducted, but will be described in a separate paper. Interviews were conducted using a semi-structured interview guide (see Additional file 3). Interviewers included two of the authors (JG and FP, both female), a research coordinator and research assistant respectively, who were trained and have experience in qualitative research. The only prior relationship interviewers would have had with participants was a possible connection for implementation or completion of the other outcome measures. Participants were not informed about any personal characteristics of the researcher, but were informed that the goal of interviews was to get their perspective of the program and experiences within it. Field notes were made by facilitators to guide the coding process and familiarize themselves with the data.

### Data collection

Process measures including recruitment, retention, and program participation (Table 2) were tracked by the research team, by the volunteer coordination agency, through metrics in the Healthy Lifestyle App, or through EMR query.

Patient-reported outcome and experience measures (Table 2) were completed at baseline and 4 months, except HbA1c which was also collected 1 year prior and 4 months after the intervention (10 months).

Individual qualitative client interviews were conducted at the university one-on-one with research staff experienced in qualitative research at participants’ 4-month mark. During their 4-month survey completion, all intervention participants were asked if they were interested in participating in an interview; interviews were then arranged over the phone.

### Data analysis

Descriptive statistics were calculated for process measures of recruitment, retention, and program participation, as well as for participant characteristics and proposed trial outcomes.

To estimate treatment effects of proposed outcome measures (PROMs and PREMs: diabetes self-efficacy, chronic condition self-efficacy, readiness to change, physical activity, patient empowerment, patient centeredness, PACIC, and HbA1c), we conducted a series of eight analysis of covariance (ANCOVA) to examine the differences between groups at 4 months. Baseline levels for the outcomes served as the covariate and randomization (control or intervention) served as the fixed factor. Between-group differences in goal attainment scores were completed using an independent-samples *t* test using a 95% confidence interval. Analyses were performed using SPSS 25. Statistics were not used to confirm differences between groups, but rather as a guide to help us estimate the effects and variances of the measures.

A qualitative descriptive method was used to organize and understand qualitative data [37]. Interviews were audio recorded, were transcribed, and were entered into NVivo 10 [38], also used for analyses. A basic coding structure was created based on interview questions. We then followed the six phases suggested for thematic analysis from Braun and Clarke [39]. First, two research team members (FP and JG) familiarized themselves with the data through review and reflection on interviews and field notes (phase 1). Initial codes were then generated (phase 2) by FP and JG, who met regularly to confirm the consistency of the coding structure and resolve any disagreements through discussion. The two worked together to search for themes (phase 3), review themes (phase 4), and define/name themes (phase 5) with regular check-in meetings and input provided from GA and LD. Data were analyzed using the constant comparative approach [40]. The resultant codes were grouped into themes using thematic analysis [39]. Phase 6 entails the report being produced as seen in this paper. As described in Lincoln and Guba [41], to increase the credibility and confirmability, triangulation was used, combining different methods of data collection, multiple perspectives, and multiple analysts [42], and to increase transferability, thick descriptions were used to describe the study sample and setting.

Meshing quantitative and qualitative methods was intended to provide a more multi-dimensional understanding of the feasibility of this study [43]. Qualitative data were used both to contextualize the quantitative data [44] and to expand on it, generating new knowledge [45, 46].

## Results

### Process measures

**Fig. 1:**
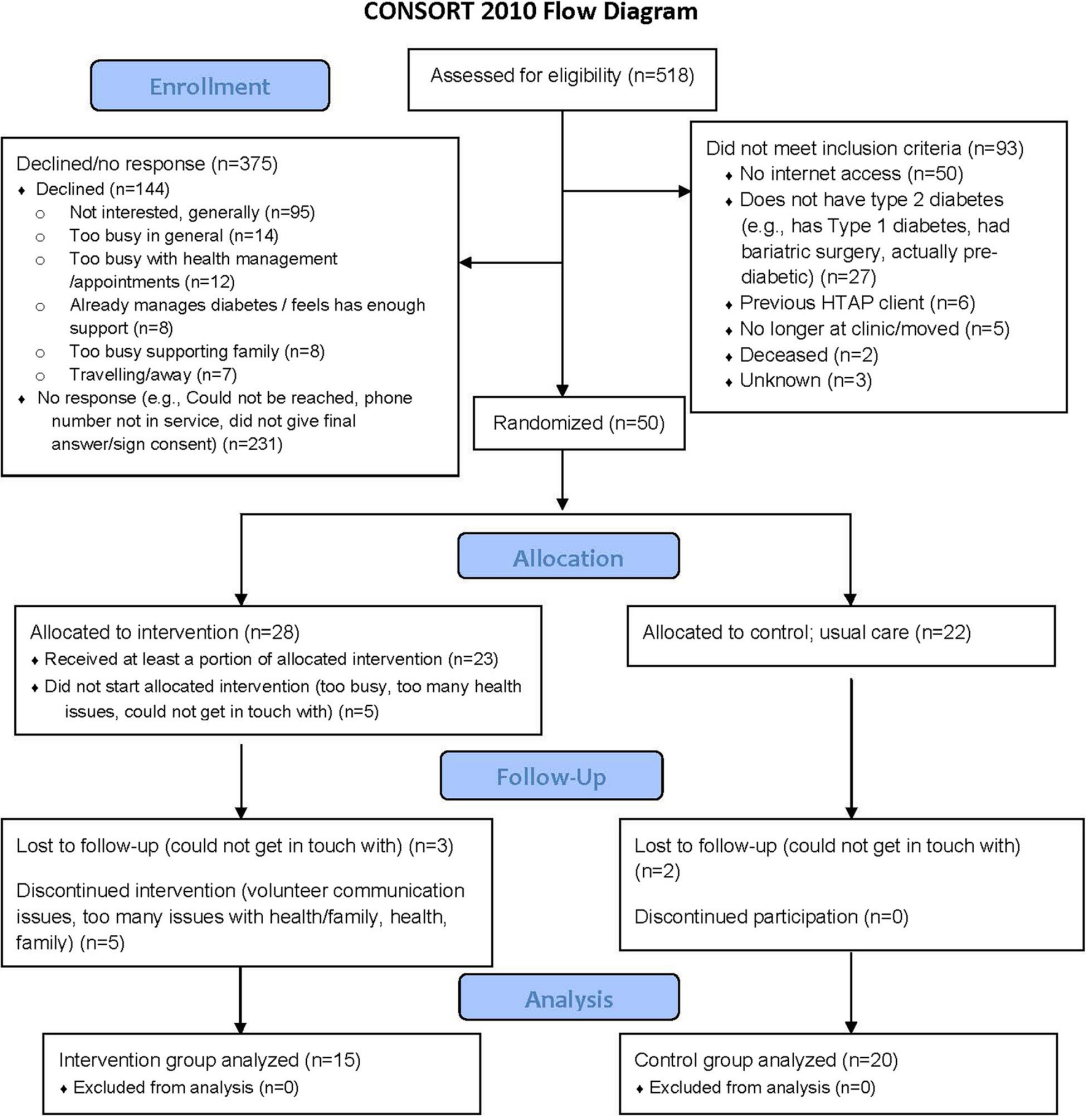
CONSORT statement image

Of 21 family physicians invited, 21 (100%) participated in the study. The initial EMR query generated a list of 518 potential participants with diabetes and hypertension. See Fig. 1 for the CONSORT flow diagram. From that list, 93 were excluded when assessed against inclusion and exclusion criteria (determined by physician screening, chart audit, or phone call with patient) primarily due to having no Internet access (*n* = 50) or reporting that they did not have type 2 diabetes (*n* = 27). Of the 425 remaining eligible and invited participants, 50 eligible participants signed consent (11.8% response rate), 144 declined, and 231 had no response (see Fig. 1 for reasons). There had been 53 clients initially randomized, as 3 were found to be ineligible after randomization and excluded at that point. Other than that issue that was rectified later with set of questions prior to randomization, the randomization process was appropriate—well-balanced between group and site (site 1, 17 intervention, 16 control; site 2, 11 intervention, 9 control). Five people from the intervention group withdrew before starting (*n* = 4) or were lost to follow-up (*n* = 1) and were not included in the main analyses. Thirty-five participants completed the study (15 of which completed the intervention; 20 were in the control group). Twelve clients participated in qualitative interviews; though interview participants were recruited based on feasibility, new themes were no longer being identified in the last interviews, indicating inductive thematic saturation [47]. Interviews took an average of 29.8 min. Demographic characteristics of participants are reported in Table 3.

**Table 3:** Participant characteristics

	Intervention group	Control group	Entire sample
*n* (% women)	26 (38.5%)	23 (52.2%)	49 (44.9%)
Age, mean (SD)	64.23 (10.07)	63.96 (6.29)	64.10 (8.43)
Age category, *n* (%)			
40–59	7 (28.0%)	6 (26.1%)	13 (27.1%)
60–79	17 (68.0%)	17 (73.9%)	34 (70.8%)
80 or over	1 (4.0%)	0 (0.0%)	1 (2.1%)
Education, *n* (%)			
At most secondary school	9 (36.0%)	8 (34.8%)	17 (35.4%)
Post-secondary or higher	16 (64.0%)	15 (65.2%)	31 (64.6%)
First language, *n* (%) English	26 (86.7%)	21 (91.3%)	47 (88.7%)
Ethnicity, *n* (%) European/white	25 (96.1%)	19 (82.6%)	44 (80.0%)
Born in Canada, *n* (%)	23 (88.5%)	16 (69.6%)	39 (79.6%)
Chronic disease diagnoses, *n* (%)			
Stroke	3 (10%)	4 (17.4)	7 (13.2%)
Cancer	5 (16.7%)	4 (17.4%)	9 (17.0%)
Chronic obstructive pulmonary disease	4 (13.3%)	2 (8.7%)	6 (11.3%)
Osteoarthritis	13 (43.3%)	9 (39.1)	22 (41.5%)
Heart disease	10 (33.3%)	4 (17.4%)	14 (26.4%)
Other	10 (33.3%)	8 (34.8%)	18 (34.0%)
Total	45 (100.0%)	31 (100.0%)	76 (100.0%)
Hospital admissions past year, mean (SD)	0.32 (.75)	0.35 (.71)	0.33 (.72)
None, *n* (%)	20 80.0%)	18 (78.3%)	38 (79.2%)
1 or more, *n* (%)	5 (20.0%)	5 (21.7%)	10 (20.8%)
Emergency visits past year, mean (SD)	0.33 (.64)	0.35 (.78)	0.34 (.70)
None, *n* (%)	18 (75.0%)	18 (78.3%)	36 (76.6%)
1 or more, *n* (%)	6 (25.0%)	5 (21.7%)	11 (23.4%)
Urgent care visit in past year, mean (SD)	0.16 (.37)	0.09 (.29)	0.13 (.33)
None, *n* (%)	21 (84.0%)	21 (91.3%)	42 (87.5%)
1 or more, *n* (%)	4 (16.0%)	2 (8.7%)	6 (12.5%)
Number of medications, mean (SD)	6.56 (4.06)	9.22 (5.46)	7.83 (4.92)
Less than 5, *n* (%)	11 (44.0%)	4 (17.4)	15 (31.3%)
5 or more, *n* (%)	14 (56.0%)	19 (82.6%)	33 (68.7%)
Falls in past year, mean (SD)	0.40 (1.61)	0.35 (.57)	0.38 (1.21)
None, *n* (%)	22 (88.0%)	16 (69.6%)	38 (79.2%)
1 or more, *n* (%)	3 (12.0%)	7 (30.4%)	10 (20.8%)

*SD* standard deviation

Twenty intervention clients and eight control clients completed at least some of the Healthy Lifestyle App surveys; specific questions missed included salt intake, most recent A1c, body mass index, and waist circumference. See Table 4 for survey completion details.

**Table 4:** Completion of Healthy Lifestyle App and outcome surveys

Healthy Lifestyle App survey	Total surveys completed, *n* (%)		Questions missed in completed surveys
	Int. *n* = 28	Cont. *n* = 22	
Medications	19 (67.8%)	8 (36.4%)	None
Sleep	20 (71.4%)	8 (36.4%)	None
Physical Activity	20 (71.4%)	8 (36.4%)	None
Goals	20 (71.4%)	8 (36.4%)	None
Hypertension	18 (64.2%)	8 (36.4%)	None
Nutrition	19 (67.8%)	8 (36.4%)	• Average salt intake per day (1 intervention, 1 control)
Diabetes	19 (67.8%)	8 (36.4%)	• Most recent A1c (12 intervention, 2 control) • Body mass index (2 intervention, 3 control; also miscalculated in 2 intervention) • Waist circumference (6 intervention; 2 control)
Outcome survey (baseline)	Int. *n* = 25	Cont. *n* = 23	Questions missed in completed surveys
Diabetes self-efficacy	25 (100%)	23 (100%)	• Q5 (1 intervention, 1 control) • Q8 (1 intervention)
Chronic disease self-efficacy	25 (100%)	23 (100%)	None
Readiness to change	25 (100%)	23 (100%)	None
Physical activity	20 (80.0%)	23 (100%)	• RAPA Aerobic question (5 intervention)
Patient empowerment	25 (100%)	23 (100%)	• Q4 (1 intervention, 1 control)
Patient-centeredness	25 (100%)	23 (100%)	• Q2 (1 intervention) • Q6 (1 intervention)
PACIC	25 (100%)	23 (100%)	• Q3 (1 control) • Q4 (1 control) • Q6 (2 intervention) • Q7 (1 intervention, 1 control) • Q8 (1 control) • Q9 (1 intervention, 2 control) • Q11 (2 intervention) • Q12 (3 intervention, 1 control) • Q13 (1 intervention, 2 control) • Q17 (1 control)

*Int.* intervention, *Cont.* control, *RAPA* Rapid Assessment of Physical Activity, *PACIC* Patient Assessment of Chronic Illness Care

Twenty-four community volunteers were recruited, 23 trained, and 20 assigned to clients; three trained volunteers withdrew before being assigned to any clients (two due to no contact, one with scheduling issues). Most volunteers were undergraduate or graduate students (13, 65.0%) with an interest in the field, and the remaining were foreign-trained or local professionals; some of these also had personal or family history with diabetes. Volunteers conducted 234 client encounters with 28 clients (all intervention clients and some control clients). This included 84 phone calls, 70 home/in-person visits, 11 online messages via kindredPHR, and 69 that were not recorded which type it was. Twenty-seven reports were sent to the clinic and seen by the interprofessional huddle team, as one client had withdrawn prior to that point.

### Assessment of trial outcomes

In-person data collection sessions with research staff ranged from 17 to 76 min at baseline (mean 32.9, SD = 14.4) and 24 to 78 min at 4 months (mean 40.1, SD = 13.7). At baseline, 25 intervention and 23 control clients completed at least some of the surveys. The most commonly missed survey was the RAPA Aerobic score (missed by 5 intervention participants). The survey with the most missed questions was the PACIC (10 questions missed by at least one participant). See Table 4.

Controlling for baseline scores, at 4 months, scores for most PROMs (diabetes self-efficacy, chronic disease self-efficacy, readiness for change) and all PREMs (patient empowerment, patient-centeredness, and patient assessment of chronic illness care) were better in the intervention group than the control group, yet there were few substantial differences. Physical activity was notably better, with the intervention group reporting higher levels of physical activity than the control group. See Table 5 for results.

**Table 5:** Participant outcome and experience measure scores

Outcome	Baseline		Four months		Difference between groups at 4 months	Effect size
	Int. *n* = 15	Cont. *n* = 20	Int. *n* = 15	Cont. *n* = 20		
	EMM (SD)	EMM (SD)	Mean (SD)	Mean (SD)	Mean (95% CI)	*η* ^2^
Diabetes self-efficacy^†^	7.81 (0.28)	7.16 (0.24)	7.93 (1.32)	7.06 (1.55)	0.65 (− 0.11 to 1.40)	0.09
Chronic disease self-efficacy^‡^	7.23 (0.36)	6.69 (0.31)	7.56 (1.67)	6.45 (1.80)	0.53 (− 0.44 to 1.52)	0.04
Readiness to change^§^	1.76 (0.19)	2.10 (0.16)	1.56 (0.66)	2.25 (1.02)	− 0.34 (− 0.85 to 0.17)	0.05
Physical activity^||^	4.56 (0.38)	3.48 (0.33)	4.73 (1.62)	3.35 (1.60)	1.07 (0.05 to 2.10)	0.12
Patient empowerment^#^	3.31 (0.20)	3.28 (0.17)	3.20 (0.99)	3.36 (0.61)	0.03 (0.52 to 0.57)	0.00
Patient-centeredness^††^	2.67 (0.24)	2.74 (0.20)	2.63 (1.00)	2.77 (0.83)	− 0.08 (− 0.72 to 0.56)	0.00
PACIC^‡‡^	3.34 (0.23)	3.03 (0.20)	3.22 (1.11)	3.12 (0.98)	0.31 (− 0.31 to 0.93)	0.03
	Int. *n* = 16	Cont. *n* = 16	Int. *n* = 16	Cont*.* *n* = 16		
HbA1c	7.89 (0.31)	6.86 (0.31)	7.78 (1.85)	6.98 (1.11)	1.02 (0.14 to 1.91)	0.16

*Int.* intervention group, *Cont.* control group, *EMM* estimated marginal mean, *SE* standard error, *CI* confidence interval

^†^Ranges from 1 to 10, higher values represent higher self-efficacy

^‡^Ranges from 1 to 10, higher values represent higher self-efficacy

^§^Ranges from 1 to 5, higher values represent less readiness-to-change

^||^Ranges from 1 to 7, higher values represent higher activity; > 6 labeled as suboptimal activity

^#^Ranges 1–4, higher values represent higher perceived patient empowerment from clinical team

^††^Ranges from 1 to 4, higher values represent higher patient-centeredness

^‡‡^Ranges from 1 to 10, higher scores represent higher satisfaction with chronic illness care

At 4 months, GAS scores were numerically higher (better goal attainment) in the intervention group, 60.00 (SD = 19.61), than the control group, 52.50 (SD = 26.53). Of the 114 goals set by clients, physical activity was the most common domain with 21 (18.4%), followed by diet/nutrition at 17 (14.9%), social connection with 15 (13.6%) and productivity goals with 13 (11.4%). See Table 6 for goal-related participant outcomes.

**Table 6:** Goal-related participant outcomes

	Four months		
	Int. *n* = 14	Cont. *n* = 20	Between-group difference at follow-up mean (SE) 95% confidence interval
Goal attainment scaling Mean (SD)	60.00 (19.61)	52.50 (26.53)	7.50 (7.92) *p* = .35
All 3 goals combined *n* (%)			
Exceeded expectations	7 (17.9)	5 (8.5)	
Met expectations	10 (25.6)	18 (30.5)	
Partly met expectations	14 (35.9)	20 (33.9)	
Did not meet expectations (stayed the same)	7 (17.9)	9 (15.3)	
Did not meet expectations (got worse)	1 (2.6)	7 (11.9)	
Goal domains *n* = 40, total goals set: 114	*n* (% of all goals set)		Domain examples used to categorize goals, adapted from Javadi et al. (2008)
Physical activity	21 (18.4)		Exercise more, walk more
Diet/nutrition	17 (14.9)		Eat less unhealthy foods, manage weight with diet
Social connection	15 (13.6)		Spend time with family/friends, go out and do social activities, socialize with pets
Productivity	13 (11.4)		Get work done, pursue hobbies, volunteer
Diabetes management	10 (8.8)		Overall diabetes management, control HbA1c/blood sugar
Weight loss	10 (8.8)		Weight loss (not specific to diet or exercise), BMI
Maintain health	10 (8.8)		Stay healthy, stay at home, stay independent
Medical (other than diabetes management)	8 (7.0)		Managing medical problems, see doctor, manage blood pressure or medications
Mental health	4 (3.5)		Keeping up mental faculties, memory, preventing degradation, emotional health
Rehabilitation	4 (3.5)		Manage pain, improve mobility / flexibility
Smoking/alcohol	2 (1.8)		Quit smoking, decrease alcohol intake
Other	3 (2.6)		Faith, travel, financial, caregiving

### Qualitative interview data

#### Perceived program feasibility

Volunteers worked with clients on motivation, discussion, and steps toward goals. It was sometimes difficult to initially set goals, but clients were happy when goals were achieved, and found it made them accountable. Several clients described achieving exercise goals and goals of checking blood sugar more in particular:

It was good to have the goals there and check in on them... One was checking my blood sugars more. So, that did help. I did check my blood sugars more often … It was good setting those goals. (Client 50)

Most clients did not engage with kindredPHR due to technical issues, difficulty with the technology, or worry of understanding content on their own, or because they were already using another tracking method. However, most found the Healthy Lifestyle App easy to navigate and had no problems; only a couple had problems logging in. On kindredPHR:

“I thought I was putting it in the right spot, it turned out it wasn’t; and … I had trouble, so, I just left it.” (Client 47)

Some found positive changes in care coordination, including a new awareness of what is available at the clinic and that communication to the clinic was more open with them being able to share accomplishments rather than just disease management issues. Some saw no change in working with their clinical team, primarily because they already had good relationships with their team.

I have always spoken … My feelings to any doctor that I had to deal with; so, that didn’t really change. That’s just me. (Client 56)

Volunteers provided clients with new knowledge, including community and online resources. The resources provided in Healthy Lifestyle App modules were perceived as accurate and informative, covering multiple topics. Some clients had not read the online resources, but many said they would read them later and continue to access them.

"I can access anything I want online if I have any questions about any medical condition or any symptoms you might have, we all do that. But, I found that the resources that were provided by TAPESTRY were simple to access and they were already there. It probably put me into different websites that were maybe a little bit more accurate. (Client 17)

However, some clients felt they did not fit the program, mostly as they felt they were already managing their diabetes well.

"I didn’t really use it that much, because I was already deep into it, and I’m, controlling my diet, losing weight, doing all that stuff. (Client 10)

#### Risks or safety issues

When asked about drawbacks, threats, or risks, participants were largely unable to see any. Only one participant mentioned that there is always a confidentiality risk with computers.

#### Benefits and areas for improvement

The strongest impact of the intervention that clients described was learning more about how lifestyle changes, particularly exercise, impact diabetes management. Some began carrying out those changes.

I’m a gym rat now … ..It motivated me to get off my butt and start exercising. I’ve seen what exercise can do to your blood sugar levels. It got me thinking more about … maybe that’s why I’m not feeling good, maybe I can improve this, maybe I can get the doctor off my back, saying, your A1C is up here and we want it here. (Client 13)

Several clients reported an improved understanding of diabetes and its care, as a result of engagement with both volunteers and the primary care team. They learned more about the importance of getting regular blood work, medication and its side effects, blood sugar, and the complications of diabetes.

"I didn’t realize what the complications were. So, that’s an area that now I watch … . You realize, like I said, I had no concept of what diabetes was. I just thought you took a needle and... that settled your sugar. (Client 4)

However, some clients found it had no impact on their diabetes management.

I didn’t see any improvement from taking it. It never changed my sugar, you know, … because my sugar is always high when I took it. (Client 11)

There were conflicting opinions with the program length. Some found it too long, some too short, and some just right. The same results were found with the frequency of (approximately weekly) volunteer visits: while some found that weekly was not too often, others felt it was difficult to fit into their lives.

"Sometimes it was a little bit too often because I’m not sitting at home waiting for them to contact me. I’m going fishing, I’m going kayaking, I’m going golfing … I have the grandchildren I spend a lot of time with. (Client 22)

#### Sequential explanation of quantitative results

The lack of many substantial results in trial outcomes can be understood further through the qualitative data. Clients’ understanding of the impact of the program varied greatly, with some feeling it improved their understanding of diabetes and its care, and others seeing no effect. Volunteers worked with clients on goals, but setting and achieving goals was difficult. Clients exhibited willingness to use the resources provided, but admitted that they might not have actually used them yet. The biggest impact clients described was learning how lifestyle changes like exercise helped diabetes management, and physical activity was the outcome with the most notable positive difference. With a longer intervention period, these findings may amplify.

## Discussion

Collectively, our findings suggest that the Health TAPESTRY-HC-DM intervention was feasible. A majority of those who started the program completed it, two primary care sites actively participated, and strong community support was demonstrated through the ease of recruiting well-suited volunteers. The randomization process for participants was appropriate. Recruitment and retention of volunteers and providers were high. The large majority of participants who started surveys in the Healthy Lifestyle App completed them all, with only the biometric questions more regularly skipped. Participants identified only the potential confidentiality risk when using the Internet as a risk; no safety issues were identified. From this perspective, a large-scale RCT would be possible to execute.

However, the main challenge to feasibility was participant recruitment. Of 425 eligible people invited to participate, only 50 (11.8%) enrolled and 35 (8.2%) completed the study; there was a larger attrition rate within the intervention group (11/26, 42.3% attrition) than in the control group (3/20, 15% attrition). It was a difficult sample to initially recruit with eligibility criteria including indications that they may need help with self-management (recent diagnosis, uncontrolled A1C, secondary organ damage), access to Internet, and willingness to take part, and as an intervention that requires constant participant involvement, it required a very engaged sample. The main reasons for declining to participate were general disinterest in joining the program, having no Internet access, and being too busy. We suspect that the fact that this study took place in highly resourced interprofessional clinics may have impacted both client participation and potential physician referral to this program. The Internet access condition will also likely become less of an issue as access becomes even more widely available in the future. If the recruitment challenges can be overcome, this initial study supports the need for a larger RCT to assess effectiveness. Overall, the facilitators of implementation outweighed the barriers: the intervention showed some benefit with low risk of harm.

The primary outcome of diabetes self-efficacy, and most secondary outcomes, showed some positive signals between groups, indicating the potential clinical benefit of the intervention. This result is in keeping with other studies that have shown that individual self-efficacy impacts effective diabetes self-management [48]. Specifically, self-efficacy is independently associated with healthy eating patterns and physical activity [49]. In Health TAPESTRY-HC-DM, though the overall intent was to help support clients in making positive behavior changes in all aspects of their diabetes management, the focus on client-set goals and asking what matters most to them in their lives meant that the clients themselves set the direction the intervention would take. That meant that although the diabetes self-efficacy survey measured a variety of self-care behaviors for managing diabetes (including diet, physical activity, blood sugar management, and seeing a physician) [28], clients actually worked on their personally set goals and may not have included all—or, in fact, any—of these areas. This was particularly interesting in relation to what we saw with the physical activity domain.

Physical activity was the number one goal domain set, clients described that they learned about how exercise can impact glycemic control, and preliminary effects of the intervention showed success in improving physical activity. This may indicate that we need to match the outcomes measured to the goals set, though physical activity may be unique: the impact of peer-based interventions on physical activity improvements can also be seen in the literature [50, 51].

Improving self-management was a major element of Health TAPESTRY-HC-DM. Though the definition of self-management can differ in the literature, this intervention was multi-component, individualized, intensive, and interactive. Multi-component, interactive self-management interventions such as this one have been shown to potentially lead to positive behavior change [7, 52]. While both group-based and individualized modes have similar effectiveness rates, individualized programs can sometimes tend to be more cost-prohibitive due to the cost of health professionals [53]. However, in a 2010 review, it was shown that intensive lifestyle interventions were actually very cost-effective on the spectrum of interventions used to prevent and control diabetes [54]. The current study avoids even the usual costs to the health care system of individualized programs by using community volunteers for connections to clients, and the primary health care team for follow-up with clients on provider-specific tasks. There are costs associated with coordinating a volunteer program, but due to the market value of the services they provide, studies have shown that the financial benefits of volunteers outweigh the costs, bringing a higher return on investment [55–57].

Based on qualitative data, it seemed that the intervention worked better for some people than others. Some clients felt they were already managing their diabetes well despite suboptimal HbA1c control, so it is possible that the resultant health status of those clients would not have been affected because they felt they had already adopted the healthy behaviors in question. We wondered if this intervention would work best for those who were more ready to change. Despite participants having uncontrolled HbA1c and/or complications associated with diabetes, the majority of participants self-reported in the readiness-to-change questionnaire that they were already in the maintenance stage of the different diabetes self-management tasks. However, based on clinical measures, these self-management tasks may not have been being carried out effectively. For future studies, there are multiple channels that could be taken. For example, we could attempt to pursue participants for whom the intervention would work best, for example by enrolling participants who have uncontrolled diabetes, have indicated they have behaviors that could change, and have indicated willingness to work on those behaviors. We could also adjust the Healthy Lifestyle App and volunteer training to increase patient awareness on more optimal self-management strategies in order to offer a range of strategies that may better suit varied individual needs.

### Strengths and limitations

One strength of the study was the mixed-methods data collection used to understand feasibility and potential effectiveness. While quantitative results showed the specific changes to trial outcomes, qualitative data enriched our understanding of the implementation and gave unique information about how and why impacts were or were not generated [46]. Another strength was that the web-based Healthy Lifestyle App used in the study had gone through usability testing before being used in this program [26]. A further strength was the strong and consistent volunteer engagement.

There were several limitations to this study. One major limitation was participant recruitment, as previously described. As a self-selected population, there may have been impacts on generalizability. Another limitation was the setting of an academic family practice, which may also limit generalizability to other sites. Finally, volunteer bias in our participants may have reduced the ability to make an impact, as many already felt empowered or that they were already managing their lifestyles.

### Implications for research and practice

Conducting this pilot RCT only allowed for measurement of intermediate clinical outcomes, but a larger trial would allow powering to detect health outcomes. A small effect size (*η*^*2*^ = 0.09) was obtained for the primary outcome of diabetes self-efficacy, between groups. For a future trial, we would need to enroll 110 participants (accounting 20% for dropouts; power = .80; alpha = .05). Since the current study ran for 6 months and was able to enroll 50 participants, we estimate that the time required in order to recruit the participants needed to undertake this full trial would be approximately 14 months. For this larger RCT, based on learnings from our recruitment challenges, adaptations would need to be made in order to reach out to more people who are truly struggling to self-manage their chronic conditions and are interested in starting the process to change. As a multi-component intervention, scaling up to a larger number of volunteers and clients may make it more difficult to implement the program consistently. However, experiences within other related Health TAPESTRY programs indicate the potential for success [15].

Overall, this study found that an integrated approach of community volunteers, eHealth technology, engagement with community resources, and integration of interprofessional primary health care teams has the potential to have positive effects on diabetes self-management behaviors in the right sample.

## Conclusions

This study of Health TAPESTRY-HC-DM, a complex intervention to help people with diabetes self-management, indicated feasibility and potential clinical benefit. Patient-reported outcome measures showed change in the hypothesized direction, notably with physical activity, which was also seen as a primary goal set by clients. However, we need a greater understanding of who this approach helped the most and more efficient recruitment processes in order to offer it on a larger scale to the most appropriate patient population.

## Supplementary information

**Additional file 1.** CONSORT Checklist for Pilot and Feasibility Trials.

**Additional file 2.** COREQ Checklist for Reporting Qualitative Research.

**Additional file 3.** Client Interview Guide.

## Data Availability

Data generated and analyzed during the current study are not publicly available, but are available from the corresponding author upon reasonable request.

## Abbreviations

ANCOVA: Analysis of covariance
CCM: Chronic care model
CI: Confidence interval
CONSORT: Consolidated Standards of Reporting Trials
EMM: Estimated marginal means
EMR: Electronic medical record
GAS: Goal attainment scaling
HbA1c: Hemoglobin A1C
Health TAPESTRY-HC-DM: Health Teams Advancing Patient Experience, Strengthening Quality through Health Connectors for Diabetes Management
MFHT: McMaster Family Health Team
MRP: Most responsible physician
PACIC: Patient Assessment of Chronic Illness Care (measure)
PHR: Personal health record (e.g., kindredPHR)
RCT: Randomized controlled trial
SD: Standard deviation
SE: Standard error
