# Supplementary material for: Pilot randomized controlled trial of a complex intervention for diabetes self-management supported by volunteers, technology, and interprofessional primary health care teams
Source: Pilot Feasibility Stud. 2019 Oct 27;5:118. doi: 10.1186/s40814-019-0504-8 (PMC6815451; doi:10.1186/s40814-019-0504-8)
Supplement: Supplementary file 3 — Additional file 3. Client Interview Guide. [file 40814_2019_504_MOESM3_ESM.docx]

##
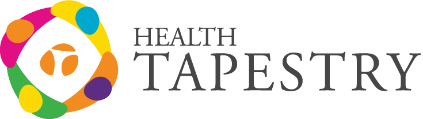


## Health TAPESTRY-HC-DM Client Interview Guide

**Thank you for taking the time to participate in this interview. We are interested in hearing about your experiences with and perceptions of the Health TAPESTRY-HC-DM program. We’ll talk about what worked well, what didn’t work as well, and what could have been done differently. We’ll start with your overall impressions and how you understand the program.**

1. **Overall Understanding of Health TAPESTRY-HC-DM**
2. What do you think is the goal or purpose of Health TAPESTRY-HC-DM?

*Probe: How would you describe the program to others?*

1. **Health TAPESTRY-HC-DM in Practice**

**Next, I’m going to ask you specifically about your participation in Health TAPESTRY-HC-DM, such as getting signed up, connecting with volunteers, completing the surveys, managing the technology, and any follow up from your clinic or others. I am interested to find out about both positive and negative experiences with the program.**

1. Thinking about your experiences in Health TAPESTRY-HC-DM:
2. Describe getting signed up for the program, and your decision to get signed up.

*Probes: What was that like? How did it go? What did you think of that? What made you want to sign up? Why do you think other people did not sign up?*

1. What was it like when you had your first volunteer visit/call scheduled, and you communicated with the Volunteer Coordinator?

*Probes: What was that like? How did that go? What did you think of that?*

1. What was it like having your weekly connection to volunteers for 4 months?

*Probes: How satisfied were you with the volunteers? How did your connect each week? What sorts of things did you do each week? How did it go? What did you think of that?*

1. What about using the TAPESTRY Healthy Lifestyle App? As a reminder, this is the online site with the surveys on it, such as Diabetes, Nutrition, Medication, and Goals.

*Probes: What did you think about the survey modules? How comfortable did you feel in entering the information? How did the volunteers support you in completing the surveys (if at all)?*

1. Specifically, what did you think about setting goals?

*Probes: Did you find it useful? How did you feel it did or did not help you reach your health goals? Was there follow-up from your health care team on your goals? How did this goal setting help you manage your own health?*

1. What did you think about the summary report you received from your responses on the Healthy Lifestyle App?

*Probes: Were you able to find the report easily? What did you find most useful about the report? What did you find least useful about it? What did you think about the resources (tip sheets) recommended on the report – did you use any? What could be improved with the resources?*

1. After your report was complete, was there any follow up from your family doctor and/or other health care professionals at your clinic (e.g. dietitian, pharmacist, OT, PT, etc.)?

*Probes*: *Did anyone from the clinic contact you based on your Health TAPESTRY results? Who (TITLE/ROLE) contacted you? What happened (e.g. phone call, follow-up appointment, home visit, etc.)? How satisfied were you with the response from your healthcare team to this information?*

1. How about using the kindredPHR (Personal Health Record)? This is the orange and white website where you can track information about your health.

*Probes: Did you log in to the PHR? If yes, what did you use it for? If no, why not? Do you plan to use the PHR after this study is over? If yes, what will you use it for? How can the PHR be improved?*

1. As a result of Health TAPESTRY-HC-DM, were you linked or referred to any community programs or services? If so, I’d like you to tell me a bit about that experience.

*Prompts: First, how were you linked to those programs? (e.g. through volunteers, clinic members, tip sheets, the EU-GENIE website, etc.) How did you use these programs or services? How did these community resources support your wellbeing? In what ways, if any, do you use community programs or services differently now than when you first became involved with Health TAPESTRY-HC-DM?*

1. Did you use EU-GENIE? As a refresher, this is the online site that volunteers may have shown you, where you make a personal map of important people in your life, and then complete a questionnaire about your interests that makes a list of local community services.

*Probes: What did you think of EU-GENIE? What worked well with EU-GENIE? What worked less well with it? Did you attend any of the community resources you were linked to? If yes, which ones? If no, why not?*

**Now I’d like to ask you a few questions about the overall experience.**

1. What do you think are the main areas for improvement for the Health TAPESTRY-HC-DM program?

*Probe: What could the program do differently?*

1. What are the main benefits of being part of Health TAPESTRY-HC-DM, for you personally, as well as for others who could be involved in the program?

*Probes: Were there any opportunities came out of your participation in the program? If so, what were they?*

1. What drawbacks, threats, or risks if any, do you think exist from taking part in the TAPESTRY-HC-DM program?

*Probe: Can you explain these?*

**Now that we’ve talked about your experiences with Health TAPESTRY-HC-DM from start-to-finish, I want to ask you about just a few other aspects of the TAPESTRY program.**

1. How has Health TAPESTRY-HC-DM affected your experiences communicating and working with members of your healthcare team, if at all? Your health care team includes: you, your health care providers (including your family doctor and other staff at the clinic, such as the nurse practitioner, dietitian, pharmacist, occupational therapist, social worker, etc.), the Health TAPESTRY-HC-DM volunteers, and your caregivers/family members.

*Probes:* *How has your relationship with your clinic changed since your involvement in the program, if at all? How has your level of confidence or satisfaction with health care at your clinic changed, if at all?*

1. How would you describe your experiences of how your health care has been coordinated over the last 4 months? By that I mean the coordination of your health care among the different members of your health care team: the volunteers, your doctor, nurses, other health care providers, community programs or services and any specialists that you see.
2. Thinking about your experience overall with Health TAPESTRY-HC-DM, in what ways, if any, did the program help you in managing your diabetes and your health in general?

*Probe*: *Has your awareness of health-related choices affecting your wellbeing changed since your involvement with the program?*

**In closing:**

1. Is there anything else you would like to add?

**Thank you so much for sharing your thoughts!**
